# Multiple Personality Disorder or Dissociative Identity Disorder: Etiology, Diagnosis, and Management

**DOI:** 10.7759/cureus.49057

**Published:** 2023-11-19

**Authors:** Mudit Saxena, Sachin Tote, Bhagyesh Sapkale

**Affiliations:** 1 Medicine, Jawaharlal Nehru Medical College, Datta Meghe Institute of Higher Education and Research, Wardha, IND; 2 Anatomy, Jawaharlal Nehru Medical College, Datta Meghe Institute of Higher Education and Research, Wardha, IND

**Keywords:** mpd, did, dissociative identity disorder management, dissociative identity disorder, multiple personality disorder

## Abstract

Dissociative identity disorder (DID), commonly known as multiple personality disorder (MPD), is a contentious mental health condition that typically arises as a result of traumatic events to help people avoid unpleasant memories. To completely comprehend the complexity and nuance of DID, this study investigates its symptomatology, diagnostic criteria, therapeutic modalities, and historical controversies. Patients with DID frequently have two or more distinct personality identities, each with its memories, characteristics, and attributes. Ten personality disorders are listed in the Diagnostic and Statistical Manual of Mental Disorders, Fifth Edition, Text Revision (DSM-5-TR), but DID, formerly known as MPD, is not one of those personality disorders. Nevertheless, myths and misunderstandings cloud our knowledge of the disease, and some critics attribute the condition's emergence to therapy rather than trauma. This study emphasizes the possibilities for recovery and fulfilling life for persons affected by DID by attempting to provide a comprehensive understanding of DID, debunk myths and misconceptions, and throw light on effective therapy methods. It accomplishes this by carefully examining the body of literature and existing studies. The DID study used a systematic strategy to obtain a thorough grasp of the causes, diagnosis, symptoms, and therapies of the disorder. It employed precise keywords and Boolean operators across four databases, prioritized current peer-reviewed English-language publications, and enforced strict exclusion standards. While admitting potential biases and limits in the databases used, the research intended to maintain methodological transparency and robustness, helping to provide an accurate and up-to-date picture of DID.

## Introduction and background

One sign of a personality disorder is an inflexible and destructive thought pattern. Personality disorders are more prevalent in clinical patients compared to the general population [1]. People may be distinguished by their name, upbringing, and personality characteristics. As long as the pathologic behavior has been present for a year or longer, all personality disorders can be diagnosed in children, except for antisocial personality disorder (ASPD) [1]. The Diagnostic and Statistical Manual of Mental Disorders, Fifth Edition, Text Revision (DSM-5-TR) lists ten personality disorders categorized into three groups. The concept of social irresponsibility is at the center of ASPD, which is characterized by exploitative, delinquent, and criminal behavior without guilt [2]. The existence of two or more separate personality identities is one sign of dissociative identity disorder (DID), along with potential neurobiological factors such as altered brain structure and function, particularly in regions like the hippocampus and amygdala, as well as disruptions in memory, identity, and emotion regulation mechanisms [3]. Well-known psychologists, including Kluft, in the year 1999 have examined the DID-in-sum idea. According to the theory, people who can dissociate are more likely to create alters with distinct names and identities, have intense traumatic experiences that distort reality, and lack external stability. As a result, it is essential for kids to self-soothe to deal with stressors [3]. DID cannot develop without these four conditions being met. Trauma spectrum disorders are a term that refers to several diseases, including post-traumatic stress disorder (PTSD), a subtype of severe depression, borderline personality disorder (BPD), and dissociative disorders (DD) [4]. DID, formerly known as MPD, is often a reaction to trauma used to help a person avoid painful memories [3].

MPD diagnosis has come under heavy fire from the initial case descriptions [5]. It was often believed that these individuals were cunning, suggestible "mythomaniacs" who could seduce gullible medical professionals. Science frequently does not provide evidence in favor of DD and dissociation. Dissociation/DD and psychological trauma, particularly cumulative and early-life trauma, are strongly associated, according to numerous lines of study [6]. Dissociation creates delusions of trauma, according to the claim made by skeptics, and DD is an artifactual state brought on by iatrogenic and sociocultural forces. DID is a chronic mental illness with a solid empirical grounding that results from neurobiological, cognitive, and interpersonal non-integration in response to excessive stress [7]. By having a lower hippocampus volume, they can be separated from other conditions that may share some of the same symptoms, such as some anxiety disorders, but are less obviously linked to stress [7]. Several myths exist regarding DID. Others think it is primarily diagnosed in North America, and some specialists may have overdiagnosed it. Some see it as a passing trend. Despite its complexity, DID is frequently considered uncommon [8]. Regardless of whether DID is declared fit to face trial, DID is legally responsible for the offense that was committed [9]. Traumatic experiences that DID patients had resulted in physiological manifestations and they had been banished from consciousness but that continued to affect their thoughts and behavior [10]. Some people mistakenly think that therapy rather than trauma is the main reason, and DID and borderline personality disorder are often confused. Concerns emerge regarding the potential negative effects of DID treatments as well.

## Review

Search methodology

**Figure 1:**
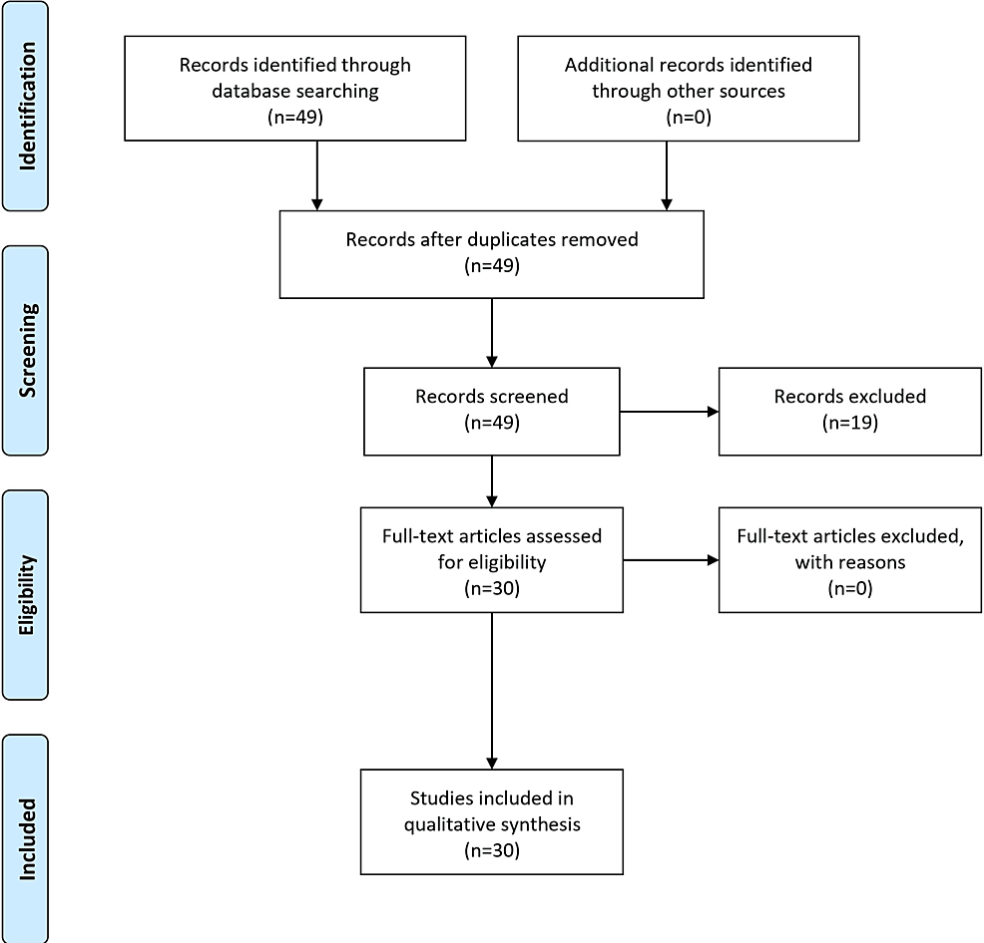
Prisma flow diagram

The goal of this narrative review article was to gain a thorough understanding of DID by looking at its causes, diagnostic standards, symptoms, and treatment options. A systematic search across four reliable databases, PubMed, MayoClinic, Google Scholar, and Cleveland Clinic, was part of the strategic methodology used in the research design. Particular search terms, like "Dissociative Identity Disorder," "Etiology," "Diagnosis," and "Symptoms," were combined with Boolean operators to guarantee the retrieval of the most relevant and up-to-date articles. By using this method, the study's rigor and usefulness were increased by trying to fully capture a broad spectrum of opinions on DID. It was intended to separate articles that discussed various facets of DID, from its causes to its therapies. Only publications that were published and written in English were taken into consideration to keep the research current and relevant. The study's credibility was ensured by favoring peer-reviewed publications, reviews, clinical guidelines, and original research pieces. Older articles and works published in languages other than English, essays, and items that superficially mentioned DID were also removed. The thorough search method turned up about 49 potential articles. This was reduced to 30 through a screening procedure based on titles and abstracts. There is a range of publication years in the references that are given. The references range in date from 1990 (Fahy's work on MPD, i.e.) to 2023 (e.g., StatPearls (Internet) references on DID). A thorough analysis of the literature on DID is made possible by including a range of publication years, guaranteeing that historical and contemporary viewpoints are considered, so 1990-2023 is the year range of the included articles for this review. The PRISMA checklist was used to evaluate the research's quality, assuring methodological robustness thoroughly. Study design, sample size, and primary outcomes were among the crucial data taken from these papers and provided an understanding of the causes, signs, symptoms, diagnoses, and available treatments for DID. This methodology for conducting research, like all others, had its drawbacks. The scope and accessibility of the chosen databases placed restrictions on the strategy. Additionally, the possibility of overlooking pertinent studies that were not listed in the chosen databases and an inherent bias in the chosen studies existed. In conclusion, our rigorous search strategy aimed to present an accurate, thorough, and current picture of DID. Every attempt was made to uphold the research's integrity, and all of the data were carefully sourced from original studies to guarantee the conclusions' utmost transparency and trustworthiness. The Prisma flow diagram is mentioned below (Figure 1).

Dissociative identity disorder etiology

Two or more distinct personality states indicate a DID. MPD (currently known as DID) often arises as a reaction to trauma to help a person escape painful memories [11]. DID is a mental health condition that manifests as the presence of two or more identities in the same patient, altering their sense of who they are and leaving them with amnesia for significant life events and private information [12]. The existence of two or more separate personality identities is one sign of DID. Each individual may have a distinctive name, past, and personality traits. There must be at least two identities for DID to exist [13]. Every identity has a unique set of fixed patterns for perceiving the world, building relationships, and considering its environment. Disorders of associative processing include loss of integration in critical processes like memory, consciousness, perception, motor control, and identity [14]. Even so, limited neuroimaging research has revealed that patients falling under this diagnostic group may exhibit specific brain activation patterns. The cingulate, insular, inferior parietal, and a neighboring region of the superior temporal cortex showed decreased activity in DID patients [15]. Making up playmates or taking part in other imaginative play cannot be the cause of a child's symptoms. The disturbance is neither caused by a general medical condition (like complex partial seizures) nor the immediate physiological effects of a substance (like blackouts or irrational behavior when under the influence) [16]. Gender dysphoria (GD) is sometimes confused with DID, a little-known psychiatric condition [17]. DID is a complicated mental illness that leaves its victims with enduring memory issues, behavioral abnormalities, and identity difficulties. There is a high correlation with early trauma, especially sexual trauma [4].

Schizotypal personality disorder can be mistakenly diagnosed as DID due to the similarities in symptoms between the two conditions [18]. The most common kind of DD not otherwise defined, also known as other defined dissociative disorders (OSDD, type 1), and DID are examples of chronic complicated DD [19]. Trauma specialists think that trauma is typically the underlying cause of both borderline symptoms and chronic complex DD [8]. DID is treated by psychodynamic psychotherapy [20].

Diagnosis of dissociative identity disorder

According to the American Psychiatric Association (2013), DID is a crippling mental illness that is associated with, among other things, alternating states of awareness and separate personality states with shifting access to autobiographical material. There is limited understanding of the neurological underpinnings of dissociative amnesia, even though it is a key symptom of DID and other DD [21]. DID is not one of the ten personality disorders classified in the DSM-5-TR [3]. Treatment for DID involves psychotherapy. Ego-state therapy, which was initially created as a form of hypnosis, has since developed into a secure therapeutic approach that can be used in conjunction with trauma processing therapies to treat DID [22]. The ideas of repression (Freud in 1895) and dissociation (Janet in 1889) are connected to dissociative illnesses [23]. The forensic psychiatry field is quite interested in dissociative amnesia. However, it is specifically DID clinically. DID link to BPD, which highlights the anomalous experiences (AE) from age five, before the emergence of possession-type dissociative identity crises in more than two decades, including clairvoyance, premonitory nightmares, and clairaudience [24]. Symptoms of DID are explained in Table 1.

**Table 1:** Symptoms of DID The above table depicts the symptoms of the DID known formerly as MPD. DID, dissociative identity disorder; MPD, multiple personality disorder

Category	Symptoms
Dissociation	Memory gaps or amnesia - feeling detached from oneself - out-of-body experiences
Identity alteration	Presence of two or more distinct personalities or identities - differences in voice, mannerisms, and even physical characteristics between identities - each identity might have its own name, personal history, and characteristics
Memory symptoms	Amnesia for personal information - inability to recall key personal events, traumatic or not - finding unfamiliar objects or writings among personal belongings
Depersonalization	Feeling that the world is strange or unreal - feeling like an outside observer of one's life
Derealization	feeling that the environment is strange or unreal (objects seem distorted; time may seem to slow down or speed up)
Emotional symptoms	Sudden emotional shifts - feeling numb or muted emotions - experiencing sudden anger, sadness, or other emotions without a clear cause
Somatic symptoms	Experiencing physical pain or other symptoms without a clear physical cause - phantom sensations, such as feeling as though one has a different body
Other symptoms	Hearing voices inside one's head (that may be perceived as coming from another identity) - engaging in behaviors that are out of character, and not remembering them later- trances or "zoning out"

DID is a complex psychological condition characterized by the presence of two or more distinct personality identities. These identities may have unique names, personal histories, and individual characteristics. People with DID often exhibit behavioral symptoms such as impulsivity, self-destructive actions, and even self-harm [25]. Mood-related manifestations can include anxiety, feelings of detachment from oneself, and mood swings [26]. On a psychological level, they might face altered consciousness, bouts of depression, and flashbacks [26]. Additionally, it is common for those affected to experience amnesia or blackouts [26]. Multiple personality states are symptoms of DID. Key symptoms include memory loss, dissociation, changing identities with distinct personalities, memory issues such as amnesia and foreign objects, depersonalization, derealization, unexpected emotional changes, physical sensations without apparent causes, and behaviors such as hearing voices [27]. The severity and combination of these symptoms vary throughout individuals. A mental health professional's assistance is necessary for both evaluation and therapy.

Management or treatment of dissociative identity disorder

Different treatments for DID are adapted to its subtleties. The key component is psychotherapy, which emphasizes trauma and encourages integration while frequently incorporating talks with the patient's many personality states [16]. CBT (cognitive behavioral therapy) treats disorders like anxiety or depression that coexist with DID by addressing negative thoughts and actions [20]. Although there is no specific treatment for DID, accompanying symptoms can be managed with the help of an attending psychiatrist using medications like antidepressants [28]. Selective serotonin reuptake inhibitors (SSRIs), serotonin and norepinephrine reuptake inhibitors (SNRIs), and tricyclic antidepressants (TCAs) are examples of antidepressants that psychiatrists may prescribe to treat depression by raising neurotransmitter levels [28]. Anxiolytics, like benzodiazepines, work by strengthening the calming effects of GABA (gamma-aminobutyric acid) to treat anxiety symptoms [28]. Family therapy promotes positive family relations and educates family members about DID [29]. Schema therapy can also be used in the case of management of the DID [30]. People with DID can and do recover with good therapy from mental health professionals who are trained in trauma and dissociation or who can receive advice from someone qualified. DID sufferers are capable of leading fulfilling lives. The treatment of DID is explained in Table 2.

**Table 2:** Treatment of DID The above table explains the management or treatment of DID. CBT, cognitive behavioral therapy; TF-CBT, trauma-focused cognitive behavioral therapy; SSRI, selective serotonin reuptake inhibitor; SNRI, serotonin and norepinephrine reuptake inhibitor; PTSD, post-traumatic stress disorder; DID, dissociative identity disorder

Treatment type	Description	Notes
Psychotherapy	Individual therapy to address trauma and promote integration. Sessions may involve talking to various alters (personality states) [16].	Considered the primary treatment for DID.
CBT	Addresses negative thought patterns and behaviors. Can be helpful for comorbid conditions like anxiety or depression [20].	They are often used in conjunction with other therapies.
TF-CBT	Utilizing a thorough and customized treatment strategy to address trauma-related negative thought patterns and behaviors, as well as effectively treating comorbid illnesses, including depression and anxiety [20].	Use of a thorough and individualized treatment strategy for TF-CBT
Hypnotherapy	Used to access and integrate traumatic memories. Can help facilitate communication between alters.	It should be approached cautiously; it is not suitable for all patients.
Medication	There's no drug specifically for DID. Atypical antipsychotics like quetiapine and olanzapine are used to treat dissociation, mood dysregulation, and hallucinations, while antidepressants like SSRIs and SNRIs are frequently recommended for depression and PTSD.	Individual reactions differ, and decisions about therapy must be discussed with a licensed healthcare provider.
Group therapy	Provides a supportive environment for sharing experiences and coping strategies.	It can be beneficial, but not all DID patients are comfortable with it.
Inpatient treatment	Sometimes necessary if there's a risk of harm to oneself or others. Suicidal ideation in people with DID requires a multifaceted approach that includes risk assessment, alter exploration and an integrated treatment plan that combines medication and therapy.	Typically short-term and followed by outpatient therapy.
Family therapy	Helps educate family members about DID. Can assist in improving dynamics and providing support.	Essential for those with a supportive family environment.

## Conclusions

MPD, currently known as DID, is still a hotly contested topic in the field of mental health. DID, which has its roots mostly in traumatic events, acts as a coping technique that enables people to put terrible memories in the past. It can have two or more personality identities, each with traits, memories, and characteristics. The DSM-5-TR emphasizes these various identities, their interaction, and the related memory lapses, dissociation, and identity shifts among its lengthy list of personality disorders. The ability to detach, significant trauma, the emergence of distinct alters, and a lack of external stability are some of the variables that contribute to DID's etiology. The strategy emphasizes reducing prejudices. This is accomplished by guaranteeing source diversity and objectivity throughout the analysis, considering both congruent and divergent opinions regarding DID's controversial aspects. However, the study recognizes some possible drawbacks, such as the biases that secondary sources inevitably contain and the excessive influence of cultural or regional perspectives. Furthermore, a heavy reliance on published works might not accurately reflect the complex reality of DID. In targeting the symptoms of DID, a variety of therapies, including psychotherapy, cognitive behavioral therapy, and hypnosis, are used; however, results may vary from person to person. The disease continues to be surrounded by skepticism, with some detractors linking its genesis more to therapeutic procedures than true trauma. Notably, it is crucial to comprehend how misconceptions and myths might hide DID's genuine nature.
